# Fertility-sparing surgery in children and adolescents with borderline ovarian tumors: a retrospective study

**DOI:** 10.1186/s13048-024-01409-0

**Published:** 2024-05-08

**Authors:** Jiayuan Zhao, Dan Wang, Ruojiao Wang, Yonglan He, Congwei Jia, Lingya Pan, Shuiqing Ma, Ming Wu, Weidi Wang, Xinghan Cheng, Junjun Yang, Yang Xiang

**Affiliations:** 1 Department of Obstetrics and Gynecology, Peking Union Medical College Hospital, Chinese Academy of Medical Science and Peking Union Medical College, Beijing, China; 2 Department of Ultrasound, Peking Union Medical College Hospital, Chinese Academy of Medical Sciences and Peking Union Medical College, Beijing, China; 3 Department of Radiology, Peking Union Medical College Hospital, Chinese Academy of Medical Sciences and Peking Union Medical College, Beijing, China; 4 Department of Pathology, Peking Union Medical College Hospital, Chinese Academy of Medical Sciences and Peking Union Medical College, Beijing, China

**Keywords:** Borderline ovarian tumor, Fertility-sparing surgery, Children, Adolescent

## Abstract

**Objective:**

To describe the characteristics of children and adolescents with borderline ovarian tumors (BOTs) and evaluate the efficacy and safety of fertility-sparing surgery (FSS) in these patients.

**Methods:**

Patients with BOTs younger than 20 years who underwent FSS were included in this study.

**Results:**

A total of 34 patients were included, with a median patient age of 17 (range, 3–19) years; 97.1% (33/34) of cases occurred after menarche. Of the patients, 82.4% had mucinous borderline tumors (MBOTs), 14.7% had serous borderline tumors (SBOTs), and 2.9% had seromucinous borderline tumor (SMBOT). The median tumor size was 20.4 (range, 8–40)cm. All patients were at International Federation of Gynecology and Obstetrics stage I and all underwent FSS: cystectomy (unilateral ovarian cystectomy, UC, 14/34, 41.2% and bilateral ovarian cystectomy, BC, 1/34, 2.9%), unilateral salpingo-oophorectomy (USO; 18/34; 52.9%), or USO + contralateral ovarian cystectomy (1/34; 2.9%). The median follow-up time was 65 (range, 10–148) months. Recurrence was experienced by 10 of the 34 patients (29.4%). One patient with SBOT experienced progression to low-grade serous carcinoma after the third relapse. Two patients had a total of four pregnancies, resulting in three live births. The recurrence rate of UC was significantly higher in MBOTs than in USO (*p* = 0.005). The 5-year disease-free survival rate was 67.1%, and the 5-year overall survival rate was 100%.

**Conclusions:**

Fertility-sparing surgery is feasible and safe for children and adolescents with BOTs. For patients with MBOTs, USO is recommended to lower the risk of recurrence.

## Introduction

Borderline ovarian tumors (BOTs) are rare in children and adolescents, with an annual incidence of 2.6 ovarian masses per 100,000 children and adolescents [1]. Epithelial tumors of the ovary account for approximately 15% of pediatric ovarian masses, most of which occur after menarche [2, 3]. In children and adolescents, BOTs account for 20–30% of ovarian epithelial tumors and less than 1% of all ovarian tumors [4, 5]. Children and adolescents are defined as younger than 20 years old according to the World Health Organization (WHO) [6].

BOTs have cytological features (nuclear atypia, high mitotic index, and epithelial hyperplasia) common to malignant ovarian tumors but lack clear stromal invasion by tumor cells. The standard treatments for BOTs include hysterectomy, bilateral salpingo-oophorectomy, and staged surgery including omentectomy, peritoneal cytology, and peritoneal biopsy of multiple sites in the abdominal cavity. In children and adolescents, fertility-sparing surgery (FSS) is recommended [7, 8]. However, FSS for early stage BOTs has been associated with an increased risk of recurrence, although no significant effect on survival was reported [9]. The recurrence and survival rates in young patients after FSS remain unclear owing to the rarity of BOTs in this population. There are few reports on children and adolescents with BOTs, with knowledge primarily derived from case reports and small single-center case series [10–12]. Data on clinical presentation, treatment, and oncologic outcomes in this population are therefore limited. Our retrospective study aimed to summarize the characteristics of pediatric patients with BOTs at our institution and evaluate the efficacy and safety of FSS for the treatment of BOTs in children and adolescents.

## Methods

The medical records of children and adolescent patients who underwent FSS for BOTs at the Peking Union Medical College Hospital (PUMCH; Beijing, China) from January 2012 to December 2022 were collected and reviewed. The inclusion criteria were as follows: (1) females aged younger than 20 years [13], (2) postoperative pathological diagnosis of BOT, and (3) patients referred to or treated at PUMCH. The exclusion criteria were as follows: (1) history of malignant tumors, (2) pathological diagnosis of benign cysts, and (3) boundary tumors associated with focal cancer. The present study was approved by the institutional ethics committee of Peking Union Medical College Hospital (K2139). Data on patient age, presenting symptoms, surgical procedures, pathology, recurrence, and current status were collected. All original pathological slides were reviewed by an author (Congwei Jia) with experience in gynecological pathology, in accordance with the 2014 World Health Organization classification of BOTs [14]. The tumors were staged according to the International Federation of Gynecology and Obstetrics (FIGO) 2014 classification [15, 16]. Tumor recurrence was confirmed pathologically following reoperation. Disease-free survival (DFS) was defined as the interval from the date of surgery to the date of first recurrence or the last follow-up. Overall survival (OS) was defined as the period from the initial surgery to death or the last follow-up. FSS was defined as preservation of the uterus and at least one ovary, and included ovarian tumor removal by unilateral cystectomy (UC), bilateral cystectomy (BC), unilateral salpingo-oophorectomy (USO), and unilateral salpingo-oophorectomy and contralateral ovarian cystectomy (USO + CC). Comprehensive staging surgery involved ascites or peritoneal lavage fluid cytology, abdominal and pelvic exploration, omentum resection, peritonectomy or multipoint biopsy, resection of other macroscopic lesions, and appendectomy. Lymphadenectomy was not routinely performed. All patients underwent initial FSS, and some underwent secondary surgery after the diagnosis of BOT was confirmed. The extent of surgery was determined as the initial surgery and any reoperation performed within two months of the initial surgery.

GraphPad Prism software version 9.0 (GraphPad Software, La Jolla, CA, USA) was used to generate the Kaplan–Meier survival curves. The Kaplan-Meier method was used to estimate DFS rates. Continuous data are expressed as the median (range). Count data are expressed as frequencies and percentages. The differences of relapse and non-relapse cases in age, tumor size, surgery procedure and follow-up time were compared using Mann–Whitney U test or one-way ANOVA test in SPSS software (version 25.0; IBM, Armonk, NY). Statistical significance was determined by *p* < 0.05.

## Results

### Clinical and pathological characteristics

**Fig. 1:**
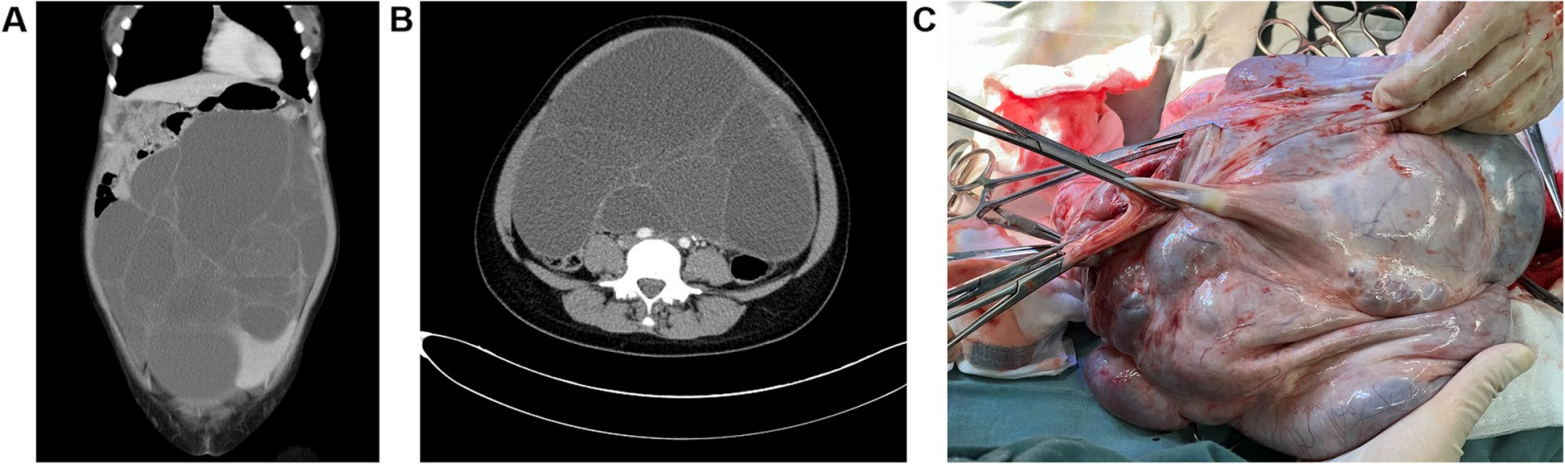
Images and surgical specimens of a representative patient with MBOT. A, the coronal image of CT; B, the axial image of CT; C, Overview of the resected mass. (MBOT, mucinous borderline tumor; CT, computed tomography)

**Fig. 2:**
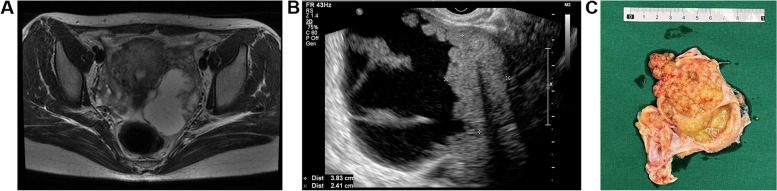
Images and surgical specimens of a representative patient with SBOT. **A**, the axial image of MRI; **B**, ultrasound image of SBOT with typical micropapillary features. **C**, Overview of the resected mass. (SBOT, serous borderline tumor; MRI, Magnetic Resonance Imaging)

Between January 2012 to December 2022, a total of 35 children and adolescents with BOTs experienced surgeries in our hospital. 34 patients conducted FSS met the inclusion criteria and were included in the current study. Eight patients first underwent FSS at other hospitals and underwent a second FSS at our hospital following recurrence. Table 1 summarizes the patient characteristics. The median age at the first surgery was 17 (range, 3–19) years, and 97.1% (33/34) of cases occurred after menarche. Of the patients, 47.1% (16/34) presented with only abdominal distension or a mass on examination, 23.5% (8/34) presented with only abdominal pain, 5.9% (2/34) presented with both abdominal distension and pain, 8.8% (3/34) presented with abnormal uterine bleeding (AUB), and 14.7% (5/34) reported no symptoms. All patients were FIGO stage I, with 76.5% (26/34) in stage IA, 5.9% (2/34) in stage IB, and 17.6% (6/34) in stage IC. The BOTs were classified into 28 (82.4%) mucinous BOTs (MBOTs, 6 with intraepithelial carcinoma), five serous BOTs (SBOTs) and one seromucinous BOT. The median size of BOTs was 20.4 (range, 8–40) cm, as determined by imaging: 3 (8.8%) were < 10 cm, 10 (29.4%) were 10–20 cm, 6 (17.6%) were 20–30 cm, 10 (29.4%) were ≥ 30 cm in size, and 5 (14.7%) did not have measurement data available. MBOTs usually present as large multi-cystic unilateral masses. And the computed tomography (CT) and surgical specimens of a MBOT patient are shown in Fig. 1A–C. The main histological features of SBOTs include the low-magnification appearance of multiple papillary structures and mild to moderate epithelial complexity. The preoperative images and intraoperative gross specimen charactered with multiple papillary structures were shown as Fig. 2A-C. Most tumors were unilateral; the left ovary was involved in 22 cases (64.7%), and the right ovary was involved in 10 cases (29.4%). Two patients (5.9%) had bilateral tumors. As for the preoperative imaging, CT examination was evaluated in 18 cases, magnetic resonance imaging (MRI) examination in 1 case, ultrasound examination in 7 cases (including 1 case also performed Positron Emission Tomography-Computed Tomography), 8 cases were not available. 69.2% (18/26) used CT as the modality of preoperative imaging evaluation. The preoperative carcinoma antigen (CA) 125 level was elevated in 58.3% (14/24) of patients in which tumor markers were assessed (range, 4.5–445.9 U/mL).

**Table 1:** Demographic and clinicopathologic patient characteristics

Characteristics		N	%
Age	Median (range), years	17(3–19)	
Menarche	Premenarchal	1	2.9%
	Menarchal	33	97.1%
Menstruation change	Hypomenorrhea	7	20.6%
	Regular	6	17.6%
	Hypomenorrhea and regula	3	8.8%
	More dysmenorrhea	2	5.9%
	No change	8	23.5%
	NA	8	23.5%
^a^FIGO stage	IA	26	76.5%
	IB	2	5.9%
	IC	6	17.6%
Histology	Mucinous	28	82.4%
	Serous	5	14.7%
	Seromucinous	1	2.9%
Clinical symptoms	Mass/distension	16	47.1%
	Pain	8	23.5%
	Mass/distension and pain	2	5.9%
	^b^AUB	3	8.8%
	No symptom	5	14.7%
Laterality	Left	22	64.7%
	Right	10	29.4%
	Bilateral	2	5.9%
^c^Tumor size (cm)	Median tumor size, range	20.4 (8–40)	
	< 10	3	8.8%
	≥ 10, < 20	10	29.4%
	≥ 20, < 30	6	17.6%
	≥ 30	10	29.4%
	NA	5	14.7%
Preoperative imaging	^d^CT	18	52.9%
	^e^PET-CT + *ultrasonography*	1	2.9%
	^f^MRI	1	2.9%
	*Ultrasonography alone*	6	17.6%
	NA	8	23.5%
^g^CA125(U/mL)	Elevated CA125	14	41.2%
	Not elevated	10	29.4%
	NA	10	29.4%
Pregnancy	Yes	2	5.9%
	No	27	79.4%
	Frozen Embryo	1	2.9%
	NA	4	11.8%
Follow-up	Median follow-up (range), months	65 (10–148)	
	Loss to follow-up	2	5.9%

^a^*FIGO* the International Federation of Obstetrics and Gynecology, ^b^*AUB* Abnormal Uterine Bleeding; ^c^Tumor size is based on preoperative imaging. ^d^*CT* Computed Tomography, ^e^*PET-CT* Positron Emission Tomography-Computed Tomography, ^f^*MRI* Magnetic resonance imaging, ^g^*CA 125* Cancer antigen 125, reference range: 0–35 U/mL

### Surgical and oncological outcomes

Table 2 shows the surgical management and prognosis of the patients. All patients underwent FSS through open (23/34; 67.6%) or laparoscopic (11/34; 32.4%) surgery: 14 patients (41.2%) underwent UC and 18 (52.9%) underwent USO; of the two patients with bilateral tumors, one underwent BC (2.9%) and one underwent USO + CC (2.9%). Seven patients (20.6%) underwent comprehensive surgical staging. Omentectomy, appendectomy, and pelvic lymphadenectomy were performed in 7 (20.6%), 14 (41.2%), and 1 (2.9%) patient, respectively. Of the 14 patients who underwent appendectomy, 13 (92.9%) had MBOT, and the one patient who underwent pelvic lymphadenectomy had MBOT with intraepithelial carcinoma. Another patient with MBOT and intraepithelial carcinoma received three cycles of paclitaxel and carboplatin after FSS. Fast frozen sections were analyzed in 20 of 34 (58.8%) cases, of which 13 (38.2%) showed consistency with paraffin pathology. In the 17 cases of MBOTs in which fast frozen sections were analyzed, 10 cases (58.8%) showed consistency with paraffin pathology. Frozen sections were obtained from three patients with SBOT, and in all cases these were consistent with paraffin pathology. Two patients were pregnant, and one patient had embryos frozen after FSS.

**Table 2:** Surgical management and prognosis of patients with borderline ovarian tumors

Surgery		N	%
Surgery approach	Open surgery	23	67.6%
	Laparoscopic surgery	11	32.4%
Surgical procedures	Unilateral ovarian cystectomy	14	41.2%
	Unilateral salpingo-oophorectomy	18	52.9%
	Bilateral ovarian cystectomy	1	2.9%
	Unilateral salpingo-oophorectomy and contralateral ovarian cystectomy	1	2.9%
	Omentectomy	7	20.6%
	Appendectomy	14	41.2%
	Pelvic lymphadenectomy	1	2.9%
Staging surgery	Yes	7	20.6%
	No	27	79.4%
fast frozen pathology	Yes (consistent with paraffin pathology)	13	38.2%
	Yes (not consistent with paraffin pathology)	7	20.6%
	No	9	26.5%
	^a^NA	5	14.7%
Recurrence	Yes	10	29.4%
	No	24	70.6%

^a^*NA* Not Available

**Fig. 3:**
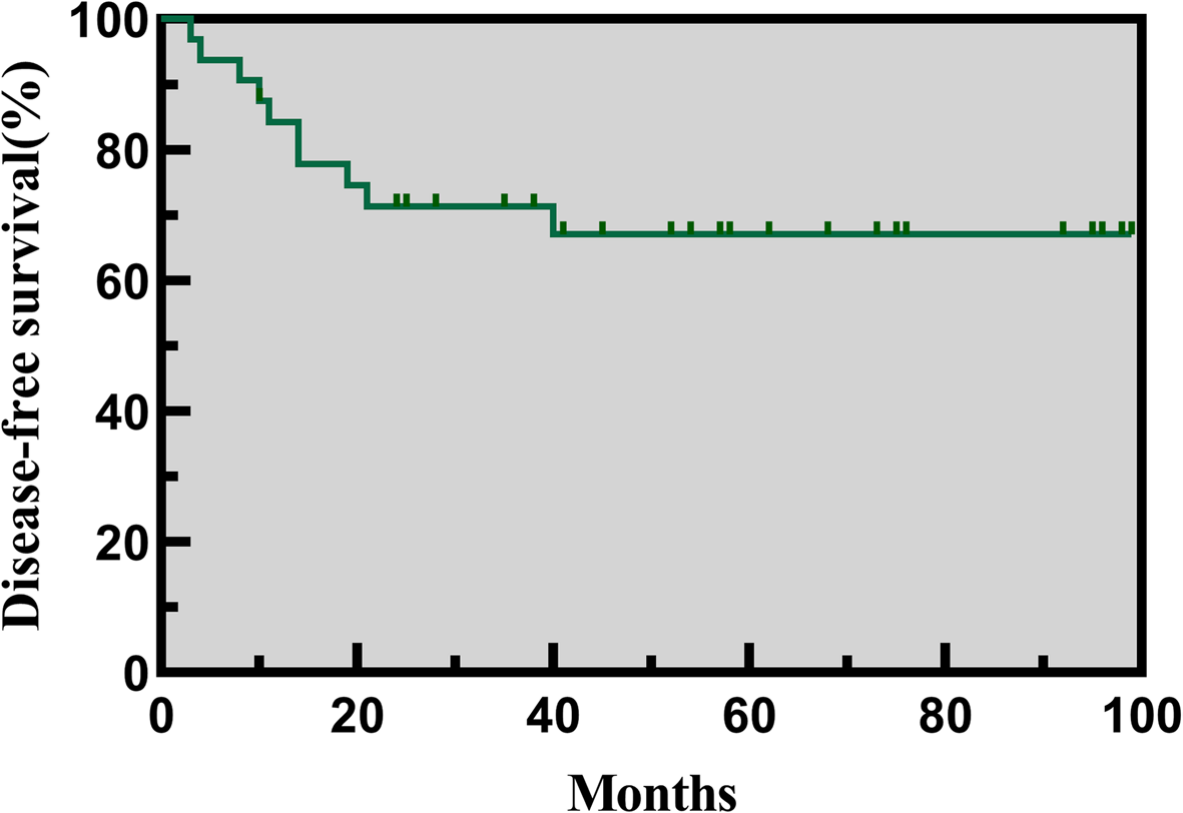
The 5-year DFS of children and adolescents with BOTs. (BOTs, borderline ovarian tumors; DFS, disease-free survival)

Table 3 shows the characteristics of patients with recurrent disease. The median follow-up period was 65(range, 10–148) months. Of the 34 patients, 10 (29.4%) experienced disease recurrence after the first surgery, including 8 patients firstly underwent FSS at other hospitals. The median recurrence time was 12.5 (range, 3–40) months. The 5-year DFS rate was 67.1% (Fig. 3) and the 5-year OS rate was 100%. All two bilateral BOTs and one SMBOT in our group experienced recurrences. One patient (Case 6) with bilateral tumors experienced three times of recurrences, with progression to low-grade serous carcinoma (LGSC) involving the rectum at the last recurrence. After three courses of doxorubicin hydrochloride and carboplatin, the patient developed incomplete bowel obstruction, which improved after conservative treatment. At the last follow-up, she was alive with disease.

**Table 3:** Characteristics of patients with recurrent disease

Cases	Age	Menarche	Side	Tumor Size (cm)	Clinical Symptoms	First Surgery	Histology	Recurrence (months)	Recurrent Surgery	Pregnancy	Progress	Following-up (months)
1	17	Yes	^a^L	19	^b^AUB	^c^LAP, ^d^UC	^e^MBOT	14	1. L, LAP, UC 2. L, LAP, UC	NO	NO	37
2	3	NO	^f^R + L	NA	Pain	LAP, ^g^RSO + LC	^h^SBOT	21	1. L, LAP, UC	^i^NA	NO	88
3	11	Yes	L	NA	Pain	^j^OS, UC	^k^SMBOT	11	1. L, LAP, ^l^USO	NO	NO	73
4	17	Yes	L	30	Mass/distension and pain	OS, UC	MBOT	10	1. L, LAP, USO 2. R, LAP, UC 3. OS, Resection of peritoneal mucinous adenofibroma + appendectomy	NO	NO	57
5	12	Yes	L	18	AUB	OS, UC	MBOT	4	1. L, LAP, USO	NO	NO	21
6	16	Yes	R + L	NA	No symptom	LAP, ^m^BC	SBOT	19	1. R + L, LAP, UC 2. R, LAP, UC 3. R + L, OS, ^o^LSO + RC + Omentectomy + Partial rectal resection and anastomosis + transverse colostomy + Appendectomy	NO	^n^LGSC	121
7	18	Yes	L	15	No symptom	LAP, UC	MBOT	14	1. L, LAP, UC + Appendectomy 2. L, LAP, UC 3. L, LAP, USO 4. R, LAP, UC 5. R, LAP, UC	Frozen Embryo	NO	148
8	18	Yes	R	NA	No symptom	OS, UC	MBOT	3	1. R, LAP, UC 2. R, LAP, USO	G2P1,1, Embryo arrest; 2. ^p^CS	NO	145
9	18	Yes	L	10.9	Mass/distension	LAP, USO	SBOT	8	1. R, LAP, UC	G3P2, 1. CS; 2. Induced abortion; 3. ^q^VD	NO	123
10	15	Yes	L	8	Pain	LAP, UC	MBOT	40	1. L, LAP, UC 2. L, LAP, USO	NO	NO	148

^a^*L* Left, ^b^*AUB* Abnormal Uterine Bleeding, ^c^*LAP* Laparoscopic Surgery, ^d^*UC* Unilateral Cystectomy, ^e^*MBOT* Mucinous Borderline ovarian tumor, ^f^*R* Right, ^g^*RSO* + *LC* Right Salpingo-Oophorectomy and left ovarian cystectomy, ^h^SBOT Serous Borderline Ovarian Tumor, ^i^*NA* Not Available, ^j^*OS* Open Surgery, ^k^*SMBOT* Seromucinous Borderline Ovarian Tumor, ^l^*USO* Unilateral salpingo-oophorectomy, ^m^*BC* Bilateral Cystectomy, ^n^*LGSC* Low-Grade Serous Carcinoma, ^o^*LSO* + *RC* Left Salpingo-Oophorectomy + Right Ovarian Cystectomy, ^p^*CS* Caesarean section, ^q^*VD* Vaginal Delivery

A comparison was conducted between relapsed and non-relapsed patients with regards to age and tumor size, revealing no statistically significant difference (age, *p* = 0.254; tumor size, *p* = 0.156). In the total group, recurrence rates are significantly higher following cystectomy compared to salpingo-oophorectomy (8/15, 53.3% vs 2/19, 10.5%, *p* = 0.007, one of bilateral BOT who performed USO + CC was counted as USO). In the subgroup of histology, the recurrence rate of UC was significantly higher in MBOTs than in USO (*p* = 0.005), but there was no statistical difference between the two procedures in SBOTs (*p* = 0.8). None of the 16 patients with MBOTs who underwent USO experienced recurrence. However, of the 12 patients with MBOTs who underwent UC, six (50%) patients relapsed. Of them, 5 patients relapsed ≥ twice. Case 5 in Table 3 was diagnosed with MBOT and first underwent UC in a local hospital, following which the tumor recurred after only four months. A USO was then conducted at our hospital, resulting in no subsequent relapses during a follow-up period of 17 months. In this study, all 10 patients with recurrence underwent an additional FSS. At the last follow-up, 60% (6/10) patients were alive without disease while 40% (4/10) alive with disease.

### Menstruation and fertility

Regarding postoperative menstrual changes (Table 1), 20.6% (7/34) of patients only experienced decreased menstrual bleeding, 17.6% (6/34) only experienced more regular menstruation, 8.8% (3/34) became both hypomenorrhea and regular, 5.9% (2/34) had more severe dysmenorrhea, 23.5% (8/34) reported no change in menstruation, and 23.5% (8/34) did not have available data. In terms of fertility (Table 3), one patient (Case 8) who experienced recurrence twice attempted to conceive 10 years after the final surgery. During the first pregnancy, she experienced embryo arrest, and the following year, she was diagnosed with antiphospholipid syndrome; this patient gave birth to a healthy boy through cesarean section in 2023. Another patient (Case 9) became pregnant less than one year after her final surgery. She gave birth by cesarean section in 2015 and vaginally in 2020, and had an induced abortion in 2016.

## Discussion

BOTs are rare among people aged younger than 20 years. To the best of our knowledge, this is the largest retrospective study conducted at a single center in children and adolescents with BOTs, involving 34 patients with BOTs aged younger than 20 years.

MBOTs and SBOTs are the two main types of BOTs. In our study, 82.4% of patients had MBOTs and 14.7% had SBOTs. The incidence of MBOTs is equivalent to or higher than that of SBOTs in Asia. In contrast, in Western countries, SBOTs represent two-thirds to three-quarters of all cases of BOTs [9, 11, 17].

Accurate preoperative diagnosis of BOTs is essential for facilitating the appropriate performance of FSS. Tumor markers have limited efficacy in diagnosing BOT and symptoms of BOTs are frequently not typical [18]. As for imaging techniques, the ultrasonography is the primary screening imaging technique in patients with ovarian masses. Ovarian crescent sign (OCS) detected by ultrasonography was defined as a rim of visible healthy ovarian tissue in the ipsilateral ovary which usually occur in benign tumor. OCS may become a simple and effective way to distinguish ovarian tumor [19]. However, several articles have shown varying probabilities of OCS occurrence in BOTs, ranging from 16% to 51.4%, with a generally low sensitivity and specificity in diagnosis of BOTs [19–22]. CT can be used to detect extra pelvic disease and estimate the FIGO stage of BOTs [18]. MRI is the preferred modality for characterization of an indeterminate ovarian mass because of its superior soft tissue resolution [23]. SBOTs present as cystic masses with papillary projections or solid masses [24]. MBOTs are characterized by microcysts that show low signal intensity on T2W MR images and exhibit reticular enhancement on contrast-enhanced T1W MR images [25]. The final diagnosis of an ovarian mass is based on the histological examination.

It is acknowledged that conservative surgery can increase the recurrence rate of BOTs [26, 27], and a study by Yokoyama et al. identified FSS as an independent risk factor for recurrence; radical surgery could reduce that risk [17]. However, Donna et al. reported no tumor-related deaths after recurrence, and a 63.6% pregnancy rate in conservative surgery cases, suggesting that FSS is a viable option for young patients, for whom fertility preservation is a major factor [28]. In our study, 3 of 5 patients with SBOTs (60%) experienced at least one relapse, while in patients with MBOTs, the recurrence rate was 21.4% (6/28). Consistent with these results, the MITO14 study reported that 53.8% (49/91) of patients with SBOTs who underwent FSS experienced at least one recurrence [29]. Song et al. reported that 16% (4/25) of patients with MBOTs relapsed after FSS [11], lower than the recurrence rate in our study; however, this may be explained by the shorter follow-up (a median follow-up period of 27 versus 65 months in our study). All patients with recurrence in our study underwent one or more additional FSS and survived.

Given that it was a retrospective study, surgery methods were determined by the comprehensive evaluation of surgeons and was discussed with the guardians and relatives. The two main FSS procedures performed at our center were cystectomy and salpingo-oophorectomy. However, recurrence rates are elevated subsequent to UC in comparison to USO in our results, especially in the subgroup of MBOTs (*p* < 0.05). A meta-analysis confirmed that cystectomy was associated with a higher recurrence rate of BOTs but no impact on the postoperative pregnancy rate [30]. MBOTs tend to be large and unilateral and contain multiple cystic spaces of varying sizes which may increase surgical difficulty and the risk of intraoperative rupture. MBOTs usually have no clear boundary with normal ovarian tissue which cystectomy could add the risk to leave some tumor cells in situ. MBOTs are also heterogeneous, with frequent co-occurrence of adenomatous, borderline, and invasive lesions [16]. Recurrence of MBOTs may indicate high invasiveness or even malignant transformation, and patients may experience multiple recurrences [31]. USO is recommended for initial treatment of MBOTs [32] and for patients with recurrent MBOTs who underwent initial cystectomy.

In our study, all of the initial recurrences of these patients occurred in the ipsilateral ovary that had been preserved in the initial surgery. The general recurrence pattern was contralateral metastasis after USO, and ipsilateral recurrence after UC. All patients with bilateral BOTs experienced recurrence, and one patient with bilateral SBOTs progressed to LGSC. Bilateral tumors are associated with invasion [33]. Despite the higher risk of recurrence after FSS, overall survival is not affected by BOT recurrence [34]. It is feasible to perform FSS again after recurrence, and the surgical procedure should be determined after full consultation with the patient and their guardians.

Intraoperative fast frozen sections are recommended to guide ovarian tumor surgery. However, the accuracy of BOT diagnosis using fast frozen sections is suboptimal [35], with the sensitivity of frozen section diagnosis for adult BOTs reported to be 62–75% [11, 36]. The diagnostic accuracy of fast frozen sections has rarely been reported in adolescents. In our study this accuracy rate was 65% (13/20); six cases (30%) were reported as mucinous cystadenoma following analysis of fast frozen sections, but diagnosed as MBOTs following paraffin pathology. Mucinous tumors are significantly associated with underdiagnosis using fast frozen sections due to their heterogeneous nature [37]. Careful observation of gross specimens before frozen pathological examination, and adequate sampling of suspicious areas, are required to optimize intraoperative MBOT diagnosis.

Long-term follow-up is crucial for children and adolescent patients after FSS [38]. Based on our data, despite the relatively short recurrence time, the recommended follow-up plan is outlined based on the experience of our center. The recommended frequency of following-up is every 3 to 6 months during the initial 5-year period, transitioning to annual examinations thereafter, with a total follow-up duration exceeding 10 years. Follow-up procedures typically involve gynecologic ultrasound and/or tumor marker assessments based on initial diagnosis, in addition to chest, abdominal, and pelvic CT and MRI scans.

Our study has several limitations. It has a small sample size and the limited representation of SBOTs may cause bias; however, this study described the clinical behaviors and evaluated the role of FSS in the largest series of BOTs in children and adolescents to date, thus contributing to our limited knowledge. This was a retrospective study that was therefore subject to selection and recall biases. And most recurrent cases were treated at different hospitals, making it difficult to ascertain the specific reasons for the surgical decisions and led to a minor loss of data. However, the rarity of BOTs in children and adolescents complicates prospective studies. A small number of cases (8/34) were initially treated in other hospitals, and then referred to our center after recurrence; thus, selection bias might exist and the surgical details and preoperative detection indicators were partially unavailable.

In conclusion, FSS is feasible and safe in children and adolescents with BOTs. For MBOTs, USO during the initial surgery is recommended to decrease the recurrence rate. Close postoperative follow-up is important to identify recurrences.

## Data Availability

No datasets were generated or analysed during the current study.

## Abbreviations

AUB: Abnormal Uterine Bleeding
BC: Bilateral Cystectomy
CA 125: Cancer Antigen 125
CT: Computed Tomography
CS: Caesarean Section
DFS: Disease-Free Survival
FIGO: The International Federation of Obstetrics and Gynecology
FSS: Fertility Sparing Surgery
LGSC: Low-Grade Serous Carcinoma
LSO: Left Salpingo-Oophorectomy
MBOT: Mucinous Borderline Ovarian Tumor
MRI: Magnetic Resonance Imaging
OS: Overall Survival
OCS: Ovarian Crescent Sign
SBOT: Serous Borderline Ovarian Tumor
SMBOT: Seromucinous Borderline Ovarian Tumor
T1W MR: T1-wighted Magnetic Resonance
T2W MR: T2-wighted Magnetic Resonance
UC: Unilateral Cystectomy
USO: Unilateral Salpingo-Oophorectomy
VD: Vaginal Delivery
USO + CC: Unilateral Salpingo-oophorectomy and Contralateral Ovarian Cystectomy
